# Analysis of sequence data to identify potential risk variants for oral clefts in multiplex families

**DOI:** 10.1002/mgg3.320

**Published:** 2017-08-09

**Authors:** Emily R. Holzinger, Qing Li, Margaret M. Parker, Jacqueline B. Hetmanski, Mary L. Marazita, Elisabeth Mangold, Kerstin U. Ludwig, Margaret A. Taub, Ferdouse Begum, Jeffrey C. Murray, Hasan Albacha‐Hejazi, Khalid Alqosayer, Giath Al‐Souki, Abdullatiff Albasha Hejazi, Alan F. Scott, Terri H. Beaty, Joan E. Bailey‐Wilson

**Affiliations:** ^1^ Computational and Statistical Genomics Branch National Human Genome Research Institute National Institutes of Health Baltimore Maryland; ^2^ National Institute of General Medical Sciences National Institutes of Health Bethesda Maryland; ^3^ Channing Division of Network Medicine Brigham and Women's Hospital Boston Massachusetts; ^4^ Harvard Medical School Cambridge Massachusetts; ^5^ Department of Epidemiology Johns Hopkins Bloomberg School of Public Health Baltimore Maryland; ^6^ Department of Oral Biology Center for Craniofacial and Dental Genetics School of Maryland Dental Medicine University of Pittsburgh Pittsburgh Pennsylvania; ^7^ Institute of Human Genetics University of Bonn Bonn Germany; ^8^ Department of Genomics Life & Brain Center University of Bonn Bonn Germany; ^9^ Department of Biostatistics Johns Hopkins Bloomberg School of Public Health Baltimore Maryland; ^10^ Department of Pediatrics University of Iowa Iowa City Iowa; ^11^ Hejazi Clinic Damascus Syrian Arab Republic; ^12^ Prime Health Clinic Jeddah Saudi Arabia; ^13^ Saudi Red Crescent Jeddah Saudi Arabia; ^14^ Al‐Eqtisad Est. Jeddah Saudi Arabia; ^15^ Center for Inherited Disease Research Johns Hopkins School of Medicine Baltimore Maryland; ^16^ Institute of Genetic Medicine Johns Hopkins School of Medicine Baltimore Maryland

**Keywords:** DNA sequence data analysis, genetic risk variants, oral clefts, rare variants, statistical genetics

## Abstract

**Background:**

Nonsyndromic oral clefts are craniofacial malformations, which include cleft lip with or without cleft palate. The etiology for oral clefts is complex with both genetic and environmental factors contributing to risk. Previous genome‐wide association (GWAS) studies have identified multiple loci with small effects; however, many causal variants remain elusive.

**Methods:**

In this study, we address this by specifically looking for rare, potentially damaging variants in family‐based data. We analyzed both whole exome sequence (WES) data and whole genome sequence (WGS) data in multiplex cleft families to identify variants shared by affected individuals.

**Results:**

Here we present the results from these analyses. Our most interesting finding was from a single Syrian family, which showed enrichment of nonsynonymous and potentially damaging rare variants in two genes: *CASP9* and *FAT4*.

**Conclusion:**

Neither of these candidate genes has previously been associated with oral clefts and, if confirmed as contributing to disease risk, may indicate novel biological pathways in the genetic etiology for oral clefts.

## Introduction

Nonsyndromic oral clefts, including cleft lip with or without cleft palate (CL/P) and cleft palate (CP) alone, are the most common craniofacial malformations in humans. The etiology of oral clefts is complex and heterogeneous with different environmental and genetic factors contributing to risk. Previous linkage and genome‐wide association studies (GWAS) have identified multiple genes and regions associated with risk for CL/P. However, it is estimated that these regions only account for 20–25% of the heritability. Improvements in sequencing technology allows us to expand our search for causal variants even further (Beaty et al. 2016). Recently, we have identified a novel, potentially damaging variant in *CDH1* in one multiplex CL/P family based on whole exome sequence (WES) data (Bureau et al. 2014). For this analysis, we used WES and whole genome sequence (WGS) data in families with distantly related affected individuals (second or third degree relationships) to identify genes containing shared rare variants.

The goal of this study was to identify novel rare variants shared at the population or the family level that may contribute to risk for oral cleft phenotypes. Our most notable finding came from a single Syrian family where we identified enrichment of nonsynonymous and potentially damaging rare variants in two genes: *CASP9* and *FAT4*. Furthermore, the *CASP9* variant was not present in any of the other affected individuals in this study, including the other Syrian families, nor is it reported in any of the major variant databases. Here we provide a summary of our results with a focus on the most interesting findings to assess the possible biological roles of these rare variants in influencing risk for oral clefts.

## Materials and Methods

### Ethical compliance

All studies were approved by the local Institutional Review/Ethics Boards and followed the tenets of the Declaration of Helsinki.

### Data collection

The multiplex cleft families studied here were originally ascertained and recruited by different studies for linkage analysis. Families were enrolled because they had at least two biological relatives affected with an apparent nonsyndromic oral cleft. Some families have been previously genotyped and included in published linkage analyses (Wyszynski et al. 2003; Field et al. 2004; Marazita et al. 2004, 2009; Schultz et al. 2004; Riley et al. 2007; Mangold et al. 2009), but the specific marker panels varied and provided sparse coverage of the genome. Families were enrolled in studies in Germany, India, the Philippines, and the Syrian Arab Republic. Each study was conducted somewhat differently, but, in general, a patient with nonsyndromic oral cleft was identified, a preliminary family history investigation revealed at least one additional affected relative existed, and the family was evaluated for potential informativeness for linkage studies. Multiplex families identified as informative were enrolled, and both affected and unaffected relatives were consented and recruited. Study participants were examined to confirm their phenotypic status, a DNA sample was collected, and, for some individuals, limited information concerning potential environmental risk factors (such as mother's smoking history during pregnancy) was available. All three types of oral clefts were identified in these families: cleft lip and palate, cleft palate only, and cleft lip only. We also obtained information on the location of the cleft (right, left, bilateral, or midline), and whether it was a complete or incomplete cleft. The specific oral cleft phenotype varied within and between families. Families were selected for this study if DNA samples were available for at least two second or third degree affected relatives who had given informed consent adequate for DNA sequence analysis. The second degree relatives included half‐sibs, avuncular, or grandparental pairs, while the third degree relatives included first cousins and great‐avuncular pairs). Some more distant affected relatives such as second cousins and first cousins once removed were also included. Due to funding constraints, only affected family members were sequenced in almost all families and parents of affected individuals were not sequenced.

For the WES portion of the study, we sequenced 108 affected individuals from 52 families (four of the families each had three affected individuals sequenced, and the remaining 48 families each had two affected individuals sequenced, four duplicate subjects for quality control, and two unrelated controls from the CEU HAPMAP population (Utah residents with Northern and Western European ancestry from the Centre d'Etude de Polymorphisme Humain (CEPH) data collection)). These multiplex families were from Syrian, Filipino, Indian, and German populations (Table 1). All samples were sequenced at the Center for Inherited Disease Research (CIDR).

**Table 1:** Number of affected individuals with nonsyndromic oral clefts^a^ and DNA sequence data

Population	Individuals (families) with WES data	Individuals (families) with WGS data
Syrian^b^	22 (10)	37 (14)
Filipino	22 (11)	76 (18)^c^
Indian	26 (12)	0
German	38 (19)	0

a Multiple affected individuals were sequenced from multiplex families.

b Three Syrian individuals from two families (total of six) have both WES and WGS data.

c Seventy affected and six unaffected individuals.

For the WGS data, there were 113 sequenced individuals (107 affected, six unaffected) from 32 families, all sequenced by Illumina (13 families with two affected individuals sequenced, two families with three affected individuals, seven families with four affected individuals, two families with three affected individuals and one unaffected, five families with five affected individuals, two families with four affected individuals and two unaffected, and one family with eight affected individuals). All of these families were from either Syrian or Filipino populations, and all of the unaffected individuals were Filipino. Table 1 shows the country of origin of these families along with individual and family counts noting the country of origin.

### Sequence data generation

#### Whole exome sequencing

Exome sequencing and genotyping was done at the CIDR. DNA sequencing was performed on an Illumina^®^ HiSeq 2500 instrument using standard protocols for a 100‐bp paired‐end run. Six samples were run per flowcell, guaranteeing >90–95% completeness at a minimum of 20× coverage.

Illumina HiSeq reads were processed through Illumina's Real‐Time Analysis (RTA) software generating base calls and corresponding quality scores. Resulting data were aligned to a reference genome with the Burrows‐Wheeler Alignment (Li and Durbin 2010) (BWA) tool creating a SAM/BAM file. Postprocessing of the aligned data includes local realignment around indels, base call quality score recalibration performed by the Genome Analysis Tool Kit (GATK) (McKenna et al. 2010; DePristo et al. 2011; Van der Auwera et al. 2013), and flagging of molecular/optical duplicates using software from the Picard program suite. Multisample variant calling was performed using GATK 2.0's Unified Genotyper. Variant quality score recalibration (VQSR) was done in GATK 2.0. CIDR required a minimum mean of 8× coverage before calling any single‐nucleotide variant (SNV), but the overall coverage averaged 84× across all exons. Further details of this process are provided in the methods section of Bureau et al. (2014).

#### Whole genome sequencing

WGS on genomic DNA samples was performed by Illumina, Inc. (San Diego, CA, USA) using TruSeq SBS v3 Reagents, HiSeq Control Software (HCS) and RTA on a HiSeq 2000 machine for real‐time image analysis and base calling. Genome assembly, genotype calling, and QC filtering was performed using tools in the CASAVA package. Multisample VCF files were generated using VCFtools (Danecek et al. 2011) and were backfilled with custom scripts to include homozygous reference genotypes and depth of coverage. Full details of the sequencing, alignment, and variant calling process are provided in the supporting information of Mathias et al. (2016).

### Sequence data filtering and annotation

#### WES data

To find potentially causal variants for oral cleft phenotypes in the WES data, we performed an initial filtering step to remove variants of low quality based on specific metrics (described below), and variants in genes with extremely high variation (Table S1) (Schmidt et al. 2013). Called variants were dropped if they failed the following quality metrics: mapping quality <30, depth <8 or depth >20,000, non‐Y SNP call rate <98%, replicate errors occurring in >1 pairs (among six duplicate subjects), monomorphic, and failing *both* the GATK VQSR filter and an in‐house machine learning metric that combines many of these QC measures to estimate the probability of being a low‐quality variant. The variant was dropped if this probability of being low quality exceeded 0.70. We then performed variant‐based counting steps at the population level and family level to identify potential risk variants for oral clefts. Here we define population as being one of the four ethnic groups (Syrian, German, Indian, and Filipino).

**Figure 1:**
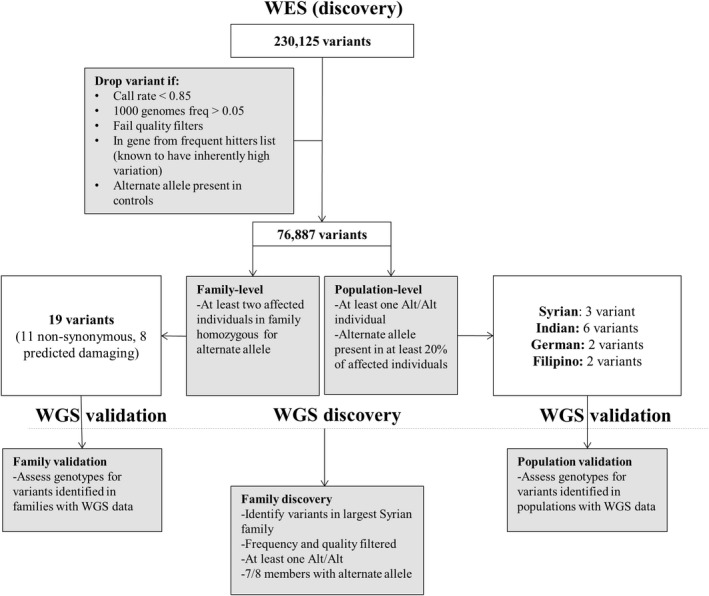
Flowchart showing single variant analysis steps for WES and WGS data.

For both family and population level analyses, we further filtered out common variants (minor allele frequency >5% in 1000 Genomes Phase I data, or in the dbSNP common variant set) and variants with an alternate allele present in either of the two controls (Fig. 1).

For the population‐specific analyses, we further screened the rare variants identifying those that were homozygous for the alternate allele in at least one case *and* at least 20% of all cases had the alternate allele in either the homozygous or heterozygous state. This criterion was chosen to allow for some within‐population heterogeneity, while enriching for potential recessive candidate variants by requiring at least one individual be homozygous for the rare allele. Since many of the families included in this study exhibit at least some degree of consanguinity, this was deemed a reasonable criterion.

For the family‐specific analysis, we identified variants that were homozygous for the alternate allele in at least two affected members of any given family. This is a relatively stringent criterion since these families had sequence data available on only two or three affected individuals. We purposely chose more stringent criteria for the family‐specific analysis because it is more likely that the risk variant is the same within the consanguineous families that made up a large proportion of the studied families as opposed to within a population. We selected homozygous variants to enrich for potentially causal recessive candidate variants since even distantly related individuals within the same family share a large number of variants by chance. Furthermore, there is a higher level of observed variant sharing than would be expected (most likely due to extensive consanguinity) in some of these families. Two families contained only one affected individual due to sequencing failure of one member and were dropped from the family‐specific analysis.

We also incorporated annotation information from wAnnovar (Wang et al. 2010; Chang and Wang 2012; Yang and Wang 2015) to assess our set of interesting genes based on potential pathogenicity, along with gene location (exonic or intronic), and predicted variant function (nonsynonymous or synonymous). Nine sources were used from wAnnovar to assess the potential pathogenicity of each variant: SIFT, Polyphen2 HDIV, Polyphen2 HVAR, LRT, Mutation Taster, Mutation Assessor, FATHMM, Radial SVM, and LR.

#### WGS data

In the WGS data, we performed both validation and discovery analyses (Fig. 1). There was very little overlap between individuals in the WES and WGS datasets (only six individuals from two families; Table 1). For our population‐specific validation analyses, we assessed genotype counts in the WGS data for variants and genes identified in the WES analyses. Validation in the population‐specific analyses was limited to the Syrian and Filipino families. Validation in the family‐specific analyses was limited to the two Syrian families that had both WES and WGS data.

As a follow‐up to the results from the single variant validation analysis for Syrian Family 1, we further analyzed the WES data to identify genes with potential compound heterozygous individuals (i.e., having two or more rare variants in the same gene). We specifically identified genes where all three affected individuals had more than one exonic, nonsynonymous variant in the WES data. We validated these results in the WGS data by assessing genotypes for all exonic, nonsynonymous variants in the genes identified in the WES analysis. We define validated genes as those with at least one WGS member who was a potential compound heterozygote.

Discovery in the WGS data was limited to the largest Syrian family (Family 1) with sequence data available on eight affected individuals. There are a total of 22 affected individuals in this large, highly consanguineous family. Almost all parents of affected individuals are consanguineous, often related through multiple paths. The eight individuals were chosen for sequencing such that they formed the most distantly related relative pairs to try to limit chance identity by descent allele sharing. After performing the same genotype quality and frequency filtering steps completed for the WES data, we identified variants with at least one family member who was homozygous for the alternate allele and at least seven of the eight family members carrying the alternate allele (this allowed for some within‐family heterogeneity, as the results from the WES analysis suggested this might be present). To reduce the number of potential risk variants to those that were more likely to be functional, we only considered exonic, nonsynonymous variants. We also performed a follow‐up analysis in the WGS data to assess rare variants in known enhancer and promoter regions of identified candidate genes.

We used the SNP and Variation Suite v8.3.4 (Golden Helix, Inc., Bozeman, MT, www.goldenhelix.com) to perform filtering, counting, annotation, and validation steps in both the WES and WGS datasets (Bozeman 2016).

## Results

### WES single variant discovery analysis

After performing the filtering steps shown in Figure 1, we used wAnnovar to assess the potential function of the most interesting variants. We also included allele frequency information from a new databases created by the Greater Middle East (GME) Variome project (Scott et al. 2016) and the Qatar Genome (Fakhro et al. 2016) for a better assessment of variants found in the Syrian population. Table 2 shows the variants identified in the family‐based analyses, along with annotation information. Tables S2–S5 show the results for the population‐specific analyses. We excluded all variants in the HLA region, as they have been shown to be highly population‐specific (Sanchez‐Mazas and Meyer 2014).

**Table 2:** Variants passing family‐specific analysis filter in WES data for all cohorts

Pop.	Fam. ID	Gene	Chr.	BP	A	R	AA	AR	RR	Location	Func.	1000G Freq.	Predicted damaging
Syrian	1	*CASP9*	1	15831171	C	T	3	0	0	Exonic	NS	–	2
	3	*HCN2*	19	603971	G	A	2	1	0	Intronic	–	0.006	–
	6	*CHRNG*	2	233408449	T	A	2	0	0	Intronic	–	0.0012	–
	7	*SLC24A4*	14	92792313	G	A	2	0	0	Exonic	NS	0.0004	2
	7	*LGMN*	14	93179134	T	C	2	0	0	Intronic	–	0.0002	–
	7	*SERPINA6*	14	94776036	A	G	2	0	0	Intronic	–	0.0002	–
	7	*HHIPL1*	14	100126748	A	G	2	0	0	Intronic	–	0.0006	–
	9	*PTGDR*	14	52734696	A	G	2	0	0	Exonic	NS	0.0006	0
	10	*FCHO1*	19	17889669	A	G	2	0	0	Exonic	NS	–	3
	10	*SUMO3*	21	46228597	T	C	2	0	0	Intronic	–	–	–
	10	*FTCD*	21	47572892	G	A	2	0	0	Exonic	NS	–	1
German	7	*MYO16*	13	109792825	T	C	2	0	0	Exonic	NS	0.0058	0
	7	*PRCD*	17	74534592	C	A	2	0	0	Upstream	–	0.0002	–
	10	*PALM*	19	740436	A	G	2	0	0	Exonic	NS	0.0018	0
	12	*CHAC1*	15	41245692	G	A	2	0	0	Exonic	NS	0.0008	1
	20	*HMHA1*	19	1081558	A	G	2	0	0	Exonic	NS	–	7
Indian	60	*DGKQ*	4	967071	A	G	2	0	0	Exonic	NS	0.027	5
Filipino	8	*TNK2*	3	195595358	T	A	2	0	0	Exonic	NS	0.0004	4
	10	*HLA‐DPA2*	6	33059894	G	A	2	0	0	Intergenic	–	0.013	–

We show the family ID for each population (Fam. ID). For each variant we give the gene name, chromosome (Chr.), base pair (BP), alternate allele (A), reference allele (R), number of individuals homozygous for the alternate allele (AA), number of heterozygous individuals (AR), number of individuals homozygous for the reference allele (RR), the gene location (Location), the function of the variant if it is exonic (NS, nonsynonymous; S, synonymous), the frequency of the alternate allele for all populations in 1000 Genomes (1000G Freq.), the frequency from the Greater Middle East Variome Project (GME Freq.), and the number of sources that predict the base pair change to be damaging out of the nine present in wAnnovar. The dashes (–) represent the following: Func. column: variants in non‐exonic regions with no defined function; 1000 Freq column: Not present in 1000 Genomes; Predicted damaging column: No pathogenicity predicted.

### WGS single variant validation analysis

We performed several validation analyses for our most interesting findings from the WES analysis in the WGS data. For the population‐specific analyses, we were able to perform validation in the Syrian and Filipino populations. All of the population‐specific discovery and validation analysis results are shown in Tables S2–S5. In the Syrian population, all of the WGS individuals were homozygous for the reference allele for the two variants identified in the *CTSL3P* gene. The variant identified in the *SYT17* gene was not present in any other subjects (Table S2). For the individuals from the Filipino population, neither of the two variants identified in the WES analysis were present in the WGS data, most likely due to low quality (Table S4).

For the family‐specific variants, we were able to perform validation in two of the Syrian families (1 and 3). As previously stated, there was little overlap between the WES and WGS individuals (Table 1), and we removed any overlapping individuals from this validation analysis.

For the eight affected individuals with WGS data in Syrian Family 1, we confirmed the homozygous genotypes observed in the three WES individuals for the nonsynonymous *CASP9* variant shown in Table 2. For the five additional affected individuals in this family, one was homozygous for the alternate allele, three were heterozygous, and one was homozygous for the reference allele (Table 3).

**Table 3:** Nonsynonymous and potentially damaging variants from Syrian Family 1 in WGS data

Gene	Chr.	BP	A	R	AA	AR	RR	Loc.	Func.	1000G Freq.	GME Freq.	QG Freq.	Predicted damaging
*CASP9*	1	15831171	C	T	4	3	1	Exonic	NS	–	–	–	2
*FAT4*	4	126367606	T	G	2	5	1	Exonic	NS	0.003	0.006	0.002	3
*FAT4*	4	126336105	G	A	2	5	1	Exonic	NS	0.002	0.007	0.002	0
*FAT4*	4	126400922	T	C	2	5	1	Exonic	NS	0.004	0.006	–	0

For each variant we give the gene name, chromosome (Chr.), base pair (BP), alternate allele (A), reference allele (R), number of individuals homozygous for the alternate allele (AA), number of heterozygous individuals (AR), number of individuals homozygous for the reference allele (RR), the gene location (Location), the function of the variant (NS, nonsynonymous; S, synonymous), the frequency of the alternate allele for all populations in 1000 Genomes (1000G Freq.), the frequency from the Greater Middle East Variome Project (GME Freq.), the frequency from the Qatar Genome data (QG Freq.), and the number of sources that predict the base pair change to be damaging out of the nine present in wAnnovar. The dashes (–) represent variants that were not present in the specific frequency database.

For Syrian Family 3, there were five individuals with WGS data, three of which were also in the WES analysis. We could not confirm the genotype for the novel variant in *HCN2* for two of the individuals from the WES analysis, because they were not included in the whole genome sequencing project. For the one WES individual who also had WGS data, the homozygous genotype for the alternate allele was confirmed. For the two individuals who were not in the WES analysis, one was heterozygous and one was homozygous for the reference allele. Thus, strong validation for this variant identified in Syrian Family 3 was not achieved due to the low number of individuals with WGS data.

### Compound heterozygous analysis (WES discovery and WGS validation)

To determine if individuals in Syrian Family 1 had any other variants potentially contributing to risk for oral clefts, we searched for genes with more than one heterozygous variant in the WES data. Such an observation may indicate that the affected individuals have two distinct deleterious alleles at the same locus. Because we do not have phase information on these individuals, we cannot rule out the possibility that the alternate alleles were inherited from the same parent as a rare haplotype. We still deem this as interesting as it could identify compound heterozygotes or the rare haplotype could itself confer increased risk for oral clefts. We defined one gene as possibly having compound heterozygotes when all three WES individuals had more than one exonic, nonsynonymous variant in that particular gene.

While there were no other exonic and/or predicted deleterious rare variants identified in *CASP9*, we did identify four genes meeting our definition of compound heterozygosity (two variants in *COL7A1*, two variants in *CELSR3*, two variants in *TKT*, and three variants in *NLRP14*) (Table 4). We then performed a validation analysis of these results using the WGS data by assessing the genotypes for all exonic, nonsynonymous variants present in these four genes. No other exonic, nonsynonymous variants were found in these genes in any of the eight family members with both WES and WGS data. We were able to confirm the heterozygous genotypes for individuals with WES data in the WGS data. Two of the five additional WGS individuals were compound heterozygous for two identified genes: *COL7A1* and *TKT* (Table 4).

**Table 4:** Variants identified in the compound heterozygous analysis in Syrian Family 1 in the WES data

Gene	Chr.	BP	A	R	AA	AR	RR	Location	Function	1000G Freq.	GME. Freq.	QG Freq.	Predicted damaging
*COL7A1*	3	48602623	A	G	0	3	0	Exonic	NS	0.001	0.005	0.003	5
*COL7A1*	3	48620046	A	G	0	3	0	Exonic	NS	0.001	0.005	0.002	7
*CELSR3*	3	48677114	G	C	0	3	0	Exonic	NS	0.019	0.011	0.013	4
*CELSR3*	3	48691197	T	C	0	3	0	Exonic	NS	0.005	0.005	0.005	0
*TKT*	3	53267183	T	C	0	3	0	Exonic	NS	0.002	0.011	0.010	3
*TKT*	3	53269028	T	G	0	3	0	Exonic	NS	0.002	0.011	0.010	1
*NLRP14*	11	7060948	T	C	0	3	0	Exonic	NS	0.014	0.040	0.046	0
*NLRP14*	11	7083610	A	T	0	3	0	Exonic	NS	0.015	0.039	0.048	5
*NLRP14*	11	7083620	C	T	0	3	0	Exonic	NS	0.022	0.043	0.051	0

For each variant we give the gene name, chromosome (Chr.), base pair (BP), alternate allele (A), reference allele (R), number of individuals homozygous for the alternate allele (AA), number of heterozygous individuals (AR), number of individuals homozygous for the reference allele (RR), the gene location (Location), the function of the variant (NS, nonsynonymous; S, synonymous), the frequency of the alternate allele for all populations in 1000 Genomes (1000G Freq.), the frequency from the Greater Middle East Variome Project (GME Freq.), and the number of sources that predict the base pair change to be damaging out of the nine present in wAnnovar.

### WGS discovery and follow‐up analyses

We performed a discovery analysis for the eight individuals with WGS data in Syrian Family 1 to search for potentially damaging variants that may have been missed in the WES analysis. First, we performed the same quality and allele frequency filtering steps as in the WES discovery analysis. We then performed genotype filtering requiring at least one family member to be homozygous for the alternate allele, and for the alternate allele to be present in at least seven of the eight other family members. We did not require that all eight of the individuals carry the alternate allele given the complex and distant relatedness patterns in this family. We further filtered variants based on annotation from wAnnovar. Specifically, we selected only rare, exonic, nonsynonymous variants. Using these steps, we identified one gene, *FAT4*, with three exonic, nonsynonymous variants, one of which was predicted to be damaging by three different sources in wAnnovar (Table 3). Genotypes for the eight total variants passing our filtering steps in this WGS validation and discovery results (all from Syrian Family 1) are listed in Table 5.

**Table 5:** Genotypes for the eight individuals with WGS data in Syrian Family 1 for the WES and WGS validation and discovery results

Ind. ID	Phen.	Single variant analyses				Compound Het. analyses			
		1:15831171 (CASP9)	4:126367606 (FAT4)	4:126336105 (FAT4)	4:126400922 (FAT4)	3:48602623 (COL7A1)	3:48620046 (COL7A1)	3:53267183 (TKT)	3:53269028 (TKT)
1 (111)*	L.CL	AA	AR	AR	AR	AR	AR	AR	AR
2 (118)*	B.CL, M.CP	AA	RR	RR	RR	AR	AR	AR	AR
3 (125)*	R.CL	AA	AR	AR	AR	AR	AR	AR	AR
4 (38)	L.CL	AA	AR	AR	AR	AR	AR	AR	AR
5 (114)	B.CL	AR	AR	AR	AR	RR	RR	RR	RR
6 (129)	L.CP‐I	RR	AA	AA	AA	AR	AR	AR	AR
7 (150)	R.CL	AR	AA	AA	AA	RR	RR	RR	RR
8 (157)	L.CL, M.CP	AR	AR	AR	AR	RR	RR	RR	RR

The first three individuals (111, 118 and 125) have WES and WGS data, as indicated by the asterisk. We identify each variant using *Chromosome:Base Pair* along with the gene name in parentheses. Homozygous for the alternate allele = AA, heterozygous = AR, homozygous for the reference allele = RR. We also show the specific cleft phenotypes for each individual (Phen. column), where L. = left, R. = right, B. = bilateral, M. = midline, I = incomplete, CL = cleft lip, and CP = cleft palate.

We also performed a follow‐up analyses to determine if there were any rare variants in eight known enhancer regions and two promoter regions of *CASP9* (Table S6). Enhancers were selected from the GeneHancer database based on their gene enhancer score (>5) (Fishilevich et al. 2017). We assessed promoters that were <200 kb from the transcription start site of *CASP9* (Stelzer et al. 2016). Table S7 shows the results from this analysis. We did identify several heterozygous variants in these potential *CASP9* regulatory regions. Interestingly, the individual with the most heterozygous variants in these regions was homozygous for the reference allele. Furthermore, no regulatory region variants were identified in the affected individuals that were homozygous for the alternate allele of the nonsynonymous, exonic *CASP9* variant. This may indicate within‐family allelic heterogeneity.

## Discussion

In this study, our goal was to identify rare potential risk variants shared by distantly related individuals with oral clefts. To do this, we performed genotype and annotation filtering steps to find genes with variants that are not common in population databases such as 1000 Genomes, but for which affected individuals were enriched at both the population and family levels. We had the most power to do this in a single Syrian family, because it had the highest number of affected individuals with sequence data and the greatest overlap between WES and WGS data. Our results reflected this, as we were able to detect eight exonic, nonsynonymous variants (four passing the single variant analysis filter and four passing the compound heterozygous filter) that show extensive sharing among these affected relatives from this highly consanguineous family. Thus, one or more of these variants may contribute to risk for oral clefts in this family.

Our most intriguing finding was a nonsynonymous variant in *CASP9* that occurred in seven of the eight family members (four of whom were homozygous for the alternate allele). We identified this as our most interesting finding for several reasons. First, this allele is not present in any of the other Syrian families, 1000 Genomes, the Qatar Genome Database, or the GME Variome project database. Second, it is a nonsynonymous, exonic variant predicted to be pathogenic by two different sources in wAnnovar. Finally, there is strong evidence that apoptotic genes play a role in the etiology of oral clefts. Among many other aspects of embryonic development, apoptosis plays a crucial role in craniofacial development. Failure of apoptosis during development may result in oral clefts (Smane et al. 2013). While no other studies to date have identified variants in *CASP9*, it is directly involved in an apoptotic signaling pathway shown to result in a facial cleft phenotype in mouse models (D'Amelio et al. 2010).

Three of the other genes identified in this study contain variants known to cause severe Mendelian syndromes (*FAT4* in Van Maldergem syndrome [Alders et al. 2014, p. 4], *COL7A1* in recessive dystrophic epidermolysis bullosa [Hovnanian et al. 1997], and *TKT* in a syndrome which includes short stature, developmental delay, and congenital heart disease [Boyle et al. 2016]). While none of these syndromes include oral clefts as a key phenotype, variants in FAT4 leading to Van Maldergem syndrome can present with craniofacial abnormalities (Alders et al. 2014, p. 4). Interestingly, the one individual from Syrian Family 1 who was homozygous for the reference allele for the variant in *CASP9* was homozygous for the alternate allele at all three of the *FAT4* variants and, further, had more than one variant predicted to be pathogenic in both *COL7A1* and *TKT*. This same individual also had the highest number of rare variants (four) in enhancer and promoter regions of *CASP9*. It is also important to note that this individual had a phenotype (incomplete cleft palate) that was distinct from the other seven family members (all others had cleft lip with or without cleft palate) (Table 5). Together, this may represent within‐family heterogeneity for genetic factors contributing to risk to nonsyndromic oral clefts.

One notable limitation of our study is there were no unaffected family members with WES data nor were there any sequenced unaffected individuals in the Syrian WGS data. This was a major limitation for our population‐based analyses, as many of these populations are not well represented in available allele frequency databases (e.g., 1000 Genomes and ExAC). Therefore, any of our interesting findings at the population‐level may be population‐specific and not truly phenotype specific. While this is also a limitation in our family‐based analyses, we have partly addressed this by limiting our results to those with a higher likelihood of being functional based on multiple annotation information (exonic, nonsynonymous, with some evidence of being pathogenic).

In this analysis we have identified rare and potentially damaging variants shared by affected family members in a single Syrian family. While these candidate genes and variants need to be assessed further in the unaffected and other affected members of this family, none have been previously identified as risk factors for nonsyndromic oral clefts and may indicate novel genetic underpinnings for this phenotype.

## Conflict of Interest

There are no conflicts of interest to disclose.

## Supporting information


**Table S1.** Frequent hitter gene names.
**Table S2.** Genes with variants that passed population‐specific analysis filter in WES data in the Syrian cohort with WGS validation.
**Table S3.** Genes with variants that passed population‐specific analysis filter in WES data in the Indian cohort.
**Table S4.** Genes with variants that passed population‐specific analysis filter in WES data in the Filipino cohort with WGS validation.
**Table S5.** Genes with variants that passed population‐specific analysis filter in WES data in the German cohort.
**Table S6.** The IDs and genomic regions for the eight enhancer regions and two promoter regions for CASP9.
**Table S7.** Genotypes for the eight individuals with WGS data in Syrian Family 1 for the variants in enhancer and promoter regions of *CASP9*.
