# Supplementary material for: Analysis of sequence data to identify potential risk variants for oral clefts in multiplex families
Source: Mol Genet Genomic Med. 2017 Aug 9;5(5):570–9. doi: 10.1002/mgg3.320 (PMC5606860; doi:10.1002/mgg3.320)
Supplement: Supplementary file 1 — Table S1. Frequent hitter gene names. Table S2. Genes with variants that passed population‐specific analysis filter in WES data in the Syrian cohort with WGS validation. Table S3. Genes with variants that passed population‐specific analysis filter in WES data in the Indian cohort. Table S4. Genes with variants that passed population‐specific analysis filter in WES data in the Filipino cohort with WGS validation. Table S5. Genes with variants that passed population‐specific analysis filter in WES data in the German cohort. Table S6. The IDs and genomic regions for the eight enhancer regions and two promoter regions for CASP9. Table S7. Genotypes for the eight individuals with WGS data in Syrian Family 1 for the variants in enhancer and promoter regions of CASP9. [file MGG3-5-570-s001.docx]

**Supplementary Table I. Frequent hitter gene names.** Variants from these genes were filtered out of the analysis due to their inherent high levels of variation.

| **Frequent hitter gene names (131 total)** | | | |
| --- | --- | --- | --- |
| A2ML1 | FADS6 | MIR3615 | SLC25A19 |
| ACOX1 | FAM104A | MIR3678 | SLC39A11 |
| AKR7L | FBF1 | MIR4738 | SLC9A3R1 |
| ANKRD36 | FDXR | MRPL38 | SMA4 |
| ARMC7 | FOXJ1 | MRPS7 | SMA5 |
| ATG12P1 | GALK1 | MTND1P17 | SMIM5 |
| ATP5H | GALR2 | MUC4 | SMIM6 |
| ATP5LP6 | GPM6BP3 | MYO15B | SOX9 |
| BTBD17 | GPR142 | NAT9 | SRP68 |
| C10orf68 | GRB2 | NT5C | SSTR2 |
| C17orf77 | GRIN2C | NUP85 | SUMO2 |
| C17orf80 | GRPC5C | OR1E2 | TEN1 |
| CALM2P1 | GTF2H2B | OR2T12 | TMEM104 |
| CASKIN2 | H3F3B | OR4C46 | TPPP |
| CD300A | HID1 | OR4M2 | TRIM47 |
| CD300C | HN1 | OR5F1 | TRIM65 |
| CD300E | HRNR | OR5H6 | TSEN54 |
| CD300LB | HSFY1P1 | OR5M10 | TTYH2 |
| CD300LD | ICT1 | OTOP2 | UNC13D |
| CD300LF | IGKV20R22-4 | OTOP3 | UNK |
| CD33 | ITGB4 | PCDHB6 | USH1G |
| CDC42EP4 | KCNJ16 | PCDHGB5 | WBP2 |
| CDH12P3 | KCNJ2 | PJA2 | WDR60 |
| CDK3 | KCNJ2-AS1 | POLR3KP2 | XKR3 |
| CDR2L | KCTD2 | RAB37 | ZACN |
| CLCNKB | KIF19 | RECQL5 | ZNF100 |
| COBL | KRTAP10-11 | RNF157 | ZNF208 |
| COG1 | KRTAP4-5 | RNF157-AS1 | ZNF402P |
| CPSF4L | LINC00469 | RPL32P33 | ZNF714 |
| CR1L | LINC00511 | RPL36AP7 |  |
| DNAI2 | LINC00673 | RPL38 |  |
| DUOXA2 | LLGL2 | SAP30BP |  |
| EVPL | MAP2K6 | SDK2 |  |
| EXOC7 | MIF4GD | SLC16A5 |  |

**Supplementary Table II. Genes with variants that passed population-specific analysis filter in WES data in the Syrian cohort with WGS validation.** Alternate alleles denoted as ‘A’ and reference alleles denoted as ‘R’. For each variant we give the gene name, chromosome (Chr.), base pair (BP), alternate allele (A), reference allele (R), number of individuals homozygous for the alternate allele (AA), number of heterozygous individuals (AR), number of individuals homozygous for the reference allele (RR), the gene location (Location), the function of the variant (NS = non-synonymous, S=synonymous), the frequency of the alternate allele for all populations in 1000 Genomes (1000G Freq.), the frequency from the Greater Middle East Variome Project (GME Freq.), the frequency from the Qatar Genome data (QG Freq.), and the number of sources that predict the base pair change to be damaging out of the 9 present in wAnnovar. NP denotes variants not present in the WGS data.

| **Gene** | **Chr.** | **Pos.** | **A** | **R** | **Loc.** | **Pred. Dam.** | **GME**  **Freq.** | **QG**  **Freq.** | **1000G Freq.** | **Discovery (WES)**  **n=22** | | | | **Validation (WGS)**  **n=37** | | | |
| --- | --- | --- | --- | --- | --- | --- | --- | --- | --- | --- | --- | --- | --- | --- | --- | --- | --- |
|  |  |  |  |  |  |  |  |  |  | **AA** | **AR** | **RR** | **Miss.** | **AA** | **AR** | **RR** | **Miss.** |
| CTSL3P | 9 | 90388511 | T | C | ncRNA | . | . | . | 0.004 | 2 | 2 | 18 | **0** | 0 | 0 | 31 | 0 |
|  | 9 | 90396201 | T | C | ncRNA | . | . | . | 0.005 | 2 | 2 | 18 | 0 | 0 | 0 | 31 | 0 |
| SYT17 | 16 | 19184712 | C | T | intronic | . | . | . | . | 2 | 3 | 15 | 2 | NP | NP | NP | NP |

**Supplementary Table III. Genes with variants that passed population-specific analysis filter in WES data in the Indian cohort.** Alternate alleles denoted as ‘A’ and reference alleles denoted as ‘R’. For each variant we give the gene name, chromosome (Chr.), base pair (BP), alternate allele (A), reference allele (R), number of individuals homozygous for the alternate allele (AA), number of heterozygous individuals (AR), number of individuals homozygous for the reference allele (RR), the gene location (Location), the function of the variant (NS = non-synonymous, S=synonymous), the frequency of the alternate allele for all populations in 1000 Genomes (1000G Freq.), and the number of sources that predict the base pair change to be damaging out of the 9 present in wAnnovar.

| **Gene** | **Chr.** | **Pos.** | **A** | **R** | **Loc.** | **Pred. Dam.** | **1000G Freq.** | **Discovery (WES)**  **n=26** | | | |
| --- | --- | --- | --- | --- | --- | --- | --- | --- | --- | --- | --- |
|  |  |  |  |  |  |  |  | **AA** | **AR** | **RR** | **Miss.** |
| DGKQ | 4 | 967071 | A | G | exonic (NS) | 4 | 0.027 | 4 | 5 | 17 | 0 |
| SACS | 13 | 23903598 | A | G | UTR3 | . | 0.022 | 1 | 12 | 13 | 0 |
| KLHDC7A | 1 | 18809351 | C | G | exonic (NS) | 8 | 0.034 | 1 | 11 | 14 | 0 |
| HERC1 | 15 | 64067595 | C | G | exonic (NS) | 1 | 0.02 | 2 | 7 | 17 | 0 |
| SHOC2 | 10 | 112745535 | A | G | intronic | . | 0.022 | 1 | 10 | 15 | 0 |
| ADRA2A | 10 | 112839026 | G | A | exonic (S) | . | 0.024 | 1 | 10 | 15 | 0 |

**Supplementary Table IV. Genes with variants that passed population-specific analysis filter in WES data in the Filipino cohort with WGS validation.** Alternate alleles denoted as ‘A’ and reference alleles denoted as ‘R’. For each variant we give the gene name, chromosome (Chr.), base pair (BP), alternate allele (A), reference allele (R), number of individuals homozygous for the alternate allele (AA), number of heterozygous individuals (AR), number of individuals homozygous for the reference allele (RR), the gene location (Location), the function of the variant (NS = non-synonymous, S=synonymous), the frequency of the alternate allele for all populations in 1000 Genomes (1000G Freq.), and the number of sources that predict the base pair change to be damaging out of the 9 present in wAnnovar. NP denotes variants not present in the WGS data.

| **Gene** | **Chr.** | **Pos.** | **A** | **R** | **Loc.** | **Pred. Dam.** | **1000G Freq.** | **Discovery (WES)**  **n=22** | | | | **Validation (WGS)**  **n=70** | | | |
| --- | --- | --- | --- | --- | --- | --- | --- | --- | --- | --- | --- | --- | --- | --- | --- |
|  |  |  |  |  |  |  |  | **AA** | **AR** | **RR** | **Miss.** | **AA** | **AR** | **RR** | **Miss.** |
| TNK2 | 3 | 195595358 | T | A | exonic (NS) | 4 | 0.0004 | 4 | 0 | 16 | 2 | NP | NP | NP | NP |
| SYT17 | 16 | 19184712 | C | T | intronic | . | . | 3 | 3 | 15 | 1 | NP | NP | NP | NP |

**Supplementary Table V. Genes with variants that passed population-specific analysis filter in WES data in the German cohort.** Alternate alleles denoted as ‘A’ and reference alleles denoted as ‘R’. For each variant we give the gene name, chromosome (Chr.), base pair (BP), alternate allele (A), reference allele (R), number of individuals homozygous for the alternate allele (AA), number of heterozygous individuals (AR), number of individuals homozygous for the reference allele (RR), the gene location (Location), the function of the variant (NS = non-synonymous, S=synonymous), the frequency of the alternate allele for all populations in 1000 Genomes (1000G Freq.), and the number of sources that predict the base pair change to be damaging out of the 9 present in wAnnovar.

| **Gene** | **Chr.** | **Pos.** | **A** | **R** | **Loc.** | **Pred. Dam.** | **1000G Freq.** | **Discovery (WES)**  **n=38** | | | |
| --- | --- | --- | --- | --- | --- | --- | --- | --- | --- | --- | --- |
|  |  |  |  |  |  |  |  | **AA** | **AR** | **RR** | **Miss.** |
| FNBP4 | 11 | 47788731 | C | G | exonic (NS) | . | 0.007 | 2 | 5 | 27 | 4 |
| CCDC15 | 11 | 124910432 | G | T | intronic | . | . | 2 | 3 | 33 | 0 |

**Supplementary Table VI. The IDs and genomic regions for the eight enhancer regions and two promoter regions for CASP9.** For the enhancers, the ID shown is the GeneHancer ID. For the promoters, the ID is the Ensembl ID.

| **Regulatory Element**  **ID** | **Genomic Region** |
| --- | --- |
|  |  |
| Enhancer 1  GH01F015507 | chr1:15834206-1583634 |
| Enhancer 2  GH01F015515 | chr1:15841495-15842295 |
| Enhancer 3  GH01F015596 | chr1:15923216-15923862 |
| Enhancer 4  GH01F015583 | chr1:15910350-15912208 |
| Enhancer 5  GH01F015678 | chr1:16004854-16012850 |
| Enhancer 6  GH01F015522 | chr1:15801101-15801900 |
| Enhancer 7  GH01F015406 | chr1:15733261-15747019 |
| Enhancer 8  GH01F015522 | chr1:15848841-15854787 |
| Promoter 1  ENSR00001577216 | chr1:15852495-15854496 |
| Promoter 2  ENSR00000531161 | chr1:15850095-15852296 |

**Supplementary Table VII. Genotypes for the eight individuals with WGS data in Syrian Family 1 for the variants in enhancer and promoter regions of *CASP9*.** The first three individuals (111, 118 and 125) have WES and WGS data, as indicated by the asterisk. The shaded rows indicate the four individuals that were either heterozygous or homozygous for the reference allele for the non-synonymous, exonic CASP9 variant. We identify each variant using *Chromosome:Base Pair* along with the gene name or the name of the regulatory element described in Supplementary Table VII. We also show the frequency of the alternate allele for all populations in 1000 Genomes (1000G Freq.), the frequency from the Greater Middle East Variome Project (GME Freq.), and the frequency from the Qatar Genome data (QG Freq.). Homozygous for the alternate allele = AA, heterozygous = AR, homozygous for the reference allele = RR. We provide the specific cleft phenotype for each individual (Phen. column) where L. = left, R. = right, B. = bilateral, M. = midline, I = incomplete, CL = cleft lip, and CP = cleft palate.

| **Ind. ID** | **Phen.** | **1:15831171**  **CASP9** | **1:15834540**  **Enhancer 1** | **1:16007462**  **Enhancer 5** | **1:16007610**  **Enhancer 5** | **1:15743432**  **Enhancer 7** | **1:15852436**  **Enhancer 8** | **1:15852846**  **Enhancer 8**  **Promoter 1** |
| --- | --- | --- | --- | --- | --- | --- | --- | --- |
| 1000G Freq.  GME Freq:  GQ Freq: | | .  .  . | 0.002  .  . | .  .  . | .  .  . | .  .  . | .  .  . | 0.004  .  . |
| 1 (111)* | L.CL | AA | RR | RR | RR | RR | RR | RR |
| 2 (118)* | B.CL,  M.CP | AA | RR | RR | RR | RR | RR | RR |
| 3 (125)* | R.CL | AA | RR | RR | RR | RR | RR | RR |
| 4 (38) | L.CL | AA | RR | RR | RR | RR | RR | RR |
| 5 (114) | B.CL | AR | RR | ***AR*** | RR | RR | RR | RR |
| 6 (129) | L.CP-I | RR | RR | RR | ***AR*** | RR | ***AR*** | ***AR*** |
| 7 (150) | R.CL | AR | ***AR*** | RR | RR | ***AR*** | RR | RR |
| 8 (157) | L.CL,  M.CP | AR | RR | RR | RR | RR | RR | RR |
